# Noninvasive brain stimulation during EEG improves machine learning classi**fi**cation in chronic stroke

**DOI:** 10.21203/rs.3.rs-4809587/v1

**Published:** 2024-09-02

**Authors:** Rishishankar E. Suresh, M. S. Zobaer, Matthew J. Triano, Brian F. Saway, Nathan C. Rowland

**Affiliations:** Medical University of South Carolina; Medical University of South Carolina; Medical University of South Carolina; Medical University of South Carolina; Medical University of South Carolina

**Keywords:** chronic stroke, machine learning, electroencephalogram, noninvasive brain stimulation, transcranial direct current stimulation

## Abstract

**Background:**

In individuals with chronic stroke and hemiparesis, noninvasive brain stimulation (NIBS) may be used as an adjunct to therapy for improving motor recovery. Specific states of movement during motor recovery are more responsive to brain stimulation than others, thus a system that could auto-detect movement state would be useful in correctly identifying the most effective stimulation periods. The aim of this study was to compare the performance of different machine learning models in classifying movement periods during EEG recordings of hemiparetic individuals receiving noninvasive brain stimulation. We hypothesized that transcranial direct current stimulation, a form of NIBS, would modulate brain recordings correlating with movement state and improve classification accuracies above those receiving sham stimulation.

**Methods:**

Electroencephalogram data were obtained from 10 participants with chronic stroke and 11 healthy individuals performing a motor task while undergoing transcranial direct current stimulation. Eight traditional machine learning algorithms and five ensemble methods were used to classify two movement states (a hold posture and an arm reaching movement) before, during and after stimulation. To minimize compute times, preprocessing and feature extraction were limited to z-score normalization and power binning into five frequency bands (delta through gamma).

**Results:**

Classification of disease state produced significantly higher accuracies in the stimulation (versus sham) group at 78.9% (versus 55.6%, p < 0.000002). We observed significantly higher accuracies when classifying stimulation state in the chronic stroke group (77.6%) relative to healthy controls (64.1%, p < 0.0095). In the chronic stroke cohort, classification of hold versus reach was highest during the stimulation period (75.2%) as opposed to the pre- and post-stimulation periods. Linear discriminant analysis, logistic regression, and decision tree algorithms classified movement state most accurately in participants with chronic stroke during the stimulation period (76.1%). For the ensemble methods, the highest classification accuracy for hold versus reach was achieved using low gamma frequency (30–50 Hz) as a feature (74.5%), although this result did not achieve statistical significance.

**Conclusions:**

Machine learning algorithms demonstrated sufficiently high movement state classification accuracy in participants with chronic stroke performing functional tasks during noninvasive brain stimulation. tDCS improved disease state and movement state classification in participants with chronic stroke.

## Background

Chronic stroke affects over seven million people in the US and remains a major source of worldwide disability.^1^ Brain computer interfaces (BCIs) are a potential method to improve quality of life for affected individuals given their ability to detect stroke severity, sense ongoing motor behavior and assist with longitudinal recovery.^2–6^ To advance BCIs for chronic stroke towards clinical practice, many groups are interested in creating a simple yet flexible BCI embedded with machine learning (ML) that can be deployed at population scale. Yet it is currently unknown what neural input features and ML approaches are optimized for this task.

Electroencephalography (EEG) recordings are noninvasive and an easily accessible method of sampling brain activity. Moreover, EEG can be paired with noninvasive brain stimulation (NIBS) to enhance certain features of control signal classification. In a recent study, high-frequency repetitive transcranial magnetic stimulation (rTMS) was used to augment detection of motor-related activity by an EEG BCI in participants performing imaginary arm flexion.^7^ While mental rehearsal has evolved into the standard paradigm for BCI studies,^8–12^ particularly in those with tetraplegia, the vast majority of participants with stroke retain some level of motor capability. Accordingly, many have questioned whether ML-assisted EEG classification in stroke participants performing real movements requires modeling a substantively different parameter space. As an example, Mebarkia et al.^13^ found that layering three support vector machines (SVM) (i.e., multi-voting) was necessary to exceed 90% accuracy in classifying left versus right hand movements in 3D space, though this BCI architecture was tested exclusively in healthy participants.

The parameter space for EEG BCIs designed for stroke rehabilitation is extensive. First, the spectral content of EEG recordings in healthy controls is significantly different from that of individuals with chronic stroke.^14–16^ For example, mu (8–12 Hz) and beta (13–30 Hz) power are attenuated following stroke, yet event-related desynchronization (ERD) and synchronization (ERS) of mu and beta are frequently used to provide the feedback signal for decoding movement intention.^17–19^ For those with severe motor deficits, ERD/ERS can be effectively absent and thus alternate control signals must be identified, though it is not clear what approach should be used to select surrogate features. Second, vascular compromise gives rise to hemispheric asymmetry (i.e., ipsi- vs contralesional), and this asymmetry is reflected as imbalanced oscillatory patterns following stroke.^20–22^ Finally, as mentioned, NIBS modalities such as rTMS have been used in combination with BCIs, however rTMS must be used in a discontinuous fashion with EEG recording due to magnetic pulse-related artifacts. Whether these factors preclude ML classification of brain state during real movement is a critical question to address prior to development of a clinically accepted BCI for chronic stroke.

In this study, we aimed to test the overall hypothesis that EEG data recorded during different movement states in individuals with chronic stroke can be accurately classified using ML. We further tested whether transcranial direct current stimulation (tDCS), which can be activated continuously during EEG recording, boosts classification accuracy compared to sham stimulation. As a control, we employed an identical ML pipeline in healthy participants.

## Materials and methods

### Participants

Ten chronic stroke (CS) participants with hemiparesis and 11 healthy controls (HC) were included in this study (IRB approved by the Medical University of South Carolina, Pro#00087153). Chronic stroke was defined as greater than six months from last ischemic stroke as determined by a fellowship-trained stroke neurologist. Mean time after stroke for CS participants was 98.8 ± 36.7 months. Those lacking the ability to reach with the affected arm were excluded.

### Randomization and Blinding

Study participants were randomly assigned to either the stimulation or sham group in a single-blinded fashion. The sham group underwent a placebo procedure that applied stimulation for 30 seconds to induce a tingling sensation in the scalp, mimicking tDCS applied for 20 minutes in the active stimulation group. Research staff operating the tDCS device were aware of group assignment.

### Task and Dataset

During a single session, participants were fitted with Ag-AgCl EEG electrodes using the 10–20 International EEG system (DC115, Rhythmlink International LLC, Columbia, SC). Next, a 5-electrode, center-surround, anodal high-density (HD) transcranial direct current stimulation (tDCS) montage was arranged on the scalp contralateral to the affected arm (model 2001tE and 4×1-C3ASKU 4×1-C3ASoterix Medical; 4×1 HD-tDCS/HD-tES adaptor, Soterix Medical, Inc, Woodbridge, NJ). The central anodal tDCS electrode was positioned near the C3 or C4 EEG electrode, depending on the laterality of the upper extremity performing the task. The surrounding cathodal tDCS electrodes were positioned in a square configuration in relation to the central anode. All tDCS electrodes were placed adjacent to, but out of direct contact with, nearby EEG electrodes. Next, an Oculus Rift (Model C4-A, Menlo Park, CA) virtual reality headset was carefully fitted over the EEG-tDCS arrangement, and a wireless controller containing an accelerometer was placed in the affected hand. EEG signals were sampled at 1024 Hz (Natus^®^ Neuroworks^®^, Pleasanton, CA). An arm reaching task designed in the Unity^®^ (version 2022.2, Unity Technologies, San Francisco, CA) programming environment began with a holding position requiring a slightly outstretched upper limb to remain in place for several seconds prior to the appearance of a colored sphere. Appearance of the sphere prompted a reaching movement (range of 15 to 50 cm) by the participant to virtually ‘touch’ the sphere and return to the holding position. The duration of the holding position was randomly varied between 2–5 seconds, and the location of the sphere was varied randomly between different locations within the virtual environment to reduce learning of the task over time. In between the hold and reach periods, a 0.5 second preparatory (or ‘prep’) cue was delivered in the form of a vibratory pulse of the controller. Participants were not explicitly instructed to attend to this cue. The trial ended after 12 consecutive hold, prep, and reach cycles were completed (average trial duration for all 12 reaches was ~ 3 minutes). Each movement cycle was divided into the following states, or “epochs”: the hold epoch, occurring prior to the prep epoch, and the reach epoch occurring from initiation of movement to return to the resting position. After an initial trial during the pre-stimulation period, the tDCS system was activated and within 30 seconds reached a maximum current delivery of 2.0 mA. At 5- and 15-minutes each after tDCS activation, two additional trials of 12 reaches were performed. Next, at 20 minutes after activation of tDCS, the tDCS current was switched off. Five minutes after deactivation of tDCS, a fourth and final trial was performed. Thus, all participants performed a total of four trials of 12 reaches each. Participants in the sham control group had tDCS activated for only 30 seconds before being immediately deactivated. The HC group underwent the same randomization and procedure. The entire experiment for each participant lasted approximately an hour.

### Preprocessing and Feature Extraction

All analyses were conducted in Python 3.9 using the NumPy,^23^ SciPy,^23^ and Scikit-Learn libraries.^24^ EEG recordings were labeled according to movement state using synchronous VR data. To achieve a minimally pre-processed pipeline, resampling and filtering of the raw signal were omitted. All data were z-score normalized over the entire EEG signal for each channel per participant, i.e., signals from participants were normalized with respect to only that subject due to heterogeneity of stroke characteristics. This was performed to significantly decrease computational demand and time during ML training. Normalization was performed via the method shown in Eq. 1.0.

Equation 1: Z-score normalization equation used within a single EEG channel

$$\text{xnorm}\;=\;\frac{x-\bar{x}}{\sigma }$$

Recordings were then divided into 1-second epochs and power spectral density (PSD) was calculated for each epoch. PSDs were generated using Welch’s method, which applies the discrete Fourier transform (DFT) to several contiguous windowed subsets of the original signal. Hann windowing was used to generate windowed segments with 50% overlap, and the number of FFT segments was set to the sample rate in order to maintain a spectral resolution of 1 Hz. PSD values were subsequently binned into frequency bands as follows: delta (1–4 Hz), theta (4–8 Hz), alpha (8–12 Hz), beta (12–30 Hz), and gamma (30–50 Hz). As bands did not exceed 50 Hz, a 60 Hz notch filter was not applied. The labeled dataset was split 70:30 for training and testing with grid search cross-validation used for hyperparameter tuning. Training was performed 10 times for each model per trial and training times were recorded.

Training was performed on a 6th Gen Intel Xeon(R) Gold 6226R processor at 2.90GHz with 64 cores and 187.5 GB RAM in serial processing alongside two NVIDIA RTX A5000 GPUs.

### Machine Learning Implementation

Classification accuracy was compared among 13 different machine learning algorithms on the same data set. The following models were chosen for this study: logistic regression (LR), linear discriminant analysis (LDA), decision trees (DT), Naïve Bayes (NB), K-nearest neighbors (KNN), random forest (RF), AdaBoost, XGBoost and heuristic voting classifiers.^25–28^ LR was chosen due to its use in motor imagery classification derived from EEG signals.^29^ The LDA classifier models the distribution of each class and was included due to its ability to perform dimensionality reduction and minimize training time.^24^ DT and RF were chosen to detect complex patterns in binarized data that may lead to a higher classification accuracy. The Naïve Bayes classifier applies Bayes theorem to calculate the probability of an observation belonging to a given class based on the assumption that the data are distributed in a Gaussian manner and was included due to its minimal training time and mathematical simplicity.^24^ KNN was included to determine if movement states exhibited clustering behavior in the associated feature space as that would reveal insights beyond improved classification accuracy. The boosting algorithms XGboost and AdaBoost were included to detect the importance of incorrectly classified data points.

The hyperparameter search process for each classifier was defined in the following way: For LR, a univariate grid search was performed on the parameter *C* for values 2^*x*^ for 15 ≤ *x*< 35. For LDA, comparisons were conducted for accuracies achieved by singular value decomposition (SVD), least squares (LSQR), and eigen solvers. Although all models demonstrated similar accuracies, SVD was chosen since it does not compute the covariance matrix and therefore has a shorter run time.

For NB classification, a Gaussian implementation was used due to the Gaussian nature of epoched EEG data. A parameter representing the variance was computed using 10^*x*^ for −15 ≤ × < 0. For KNN, the number of neighbors considered was varied for 3 ≤ *x* < 10. For DT, the minimum weight fraction of each leaf node was empirically determined to be 0. A grid search was performed to optimize the minimum number of samples required to split individual nodes (varied for 2 ≤ *x* < 11) and the maximum depth allowed for trees (varied for 2 ≤ *x* < 30). For RF, the minimum number of samples per leaf was optimally determined to be 1, the method of determining the maximum samples to split a node was set to the square root of the total number of samples, and the maximum depth of each tree was set to 30. A grid search was performed to optimize the minimum number of samples required to split individual nodes (varied from 2 ≤ *x* < 5); the number of individual trees was varied over the set *5n* for 5 ≤ *n* < 21. Feature importance was calculated by taking each feature’s average depth of use and weighing the average from one relative to the other features’ depths. The earlier a feature was used in a tree, the more important it was considered. For AdaBoost, NB and DT were contrasted as base estimators, and the number of individual estimators was varied by 25*x* for 8 ≤ × ≤ 16. For XGBoost, the number of individual estimators was varied by 25*x* for 50 ≤ × ≤ 400 and max tree depth varied by 5x for 5 ≤ *x* ≤ 30. The “Hist” method as implemented by XGBoost 2.0.3^30^ was chosen for the Tree Method hyperparameter to reduce training time. For voting classifiers, an ensemble of pre-trained models for LR, LDA, DT, RF, NB, and KNN was first created. One hard voting classifier and four soft voting classifiers among these were then utilized. Soft voting classifiers used the following weight methods: uniform (“uni”) weights, weights determined by the individual models’ training set accuracy (termed “train”), uniform weights determined by the highest model accuracy (termed “hard”), and weights predetermined based upon empirical global accuracy of all models within the ensemble (termed “global”). The ensemble labeled “me” was weighted based on the mean of several selected base estimators that appeared to perform better during the initial classification tests used to classify hold versus reach. Effect weights, hyperparameters and compute times were saved for each training. Prediction results were stored as csv files labeled by electrode and feature.

### Statistical analysis

Statistical analysis was conducted using the *rstatix* package for R (version 4.3.1).^31^ Combining data by two to three features for each model (e.g., grouping all electrodes and frequencies) resulted in groups sufficiently large to satisfy the central limit theorem and were therefore treated as parametric data. For comparisons between multiple groups, one-way ANOVA was used. Sphericity was confirmed using Mauchly’s test. Data visualizations were generated using the *ggpubr* package.^32^ The default threshold for significance was set at p < 0.05 for all tests.

## Results

Twenty-one participants (CS = 10; HC = 11) completed EEG recordings while performing a VR-guided motor task. CS and HC groups did not differ with regard to sex (χ^2^[_1_] < 0; p > 0.05). CS participants (63.3 ± 10.2 years) had a higher mean age than HC participants (46.3 ± 11.3 years; t[_18_] = 2.31, *p* = 0.0319). Six CS participants had left-sided infarct while four had right-sided infarct. From this data set, a total of 4030 models were trained with a total compute time of 103 hours.

We used 13 different ML algorithms (see Methods) to explore the effect of classification accuracy on disease state, stimulation state, movement states, frequency band, time period and electrode location. Time periods examined were pre-stimulation, intra-stimulation at 5 and 15 minutes after stimulation began, and post-stimulation, hereafter referred to as ‘Pre’, ‘Intra5’, ‘Intra15’ and ‘Post’. Features were tested in limited combinations to 1) reduce the exponential increase in model expansion and 2) to narrow clinical interpretability of the parameter space. Note that all accuracies depicted are the classification accuracies on the validation set; no samples in the validation set were used to train any of the models.

**Figure 1:**
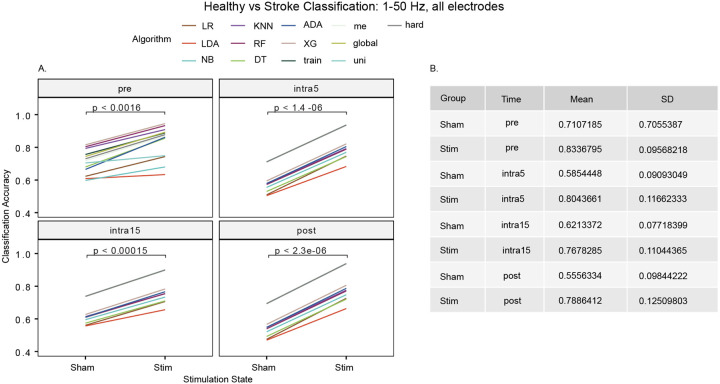
Classification of disease state (healthy versus stroke) using all frequencies from 1–50 Hz and all electrodes. Across all four stimulation time periods, we observed significantly increased accuracies after stimulation when compared to sham.

To investigate the baseline model accuracy of discriminating between healthy and chronic stroke participants, as a control we compared each algorithm using all electrodes, frequency bands, and movement states grouped together. We observed that the mean accuracy of classifying HC and CS participants was 71.1% for the sham group and 83.4% for the stim group during the pre-stimulation time period, likely due to the increased hemispheric asymmetry evident in the recordings relative to healthy controls (p < 0.0016). A higher mean classification accuracy for stim versus sham groups persisted throughout all intra- and post-stimulation time periods and was greatest at the intra5 time period (stim: 80.4 ± 11.7% versus sham: 58.5 ± 9.1%; t[_24_] = 6.3653 p < 1.4e-6, Fig. 1).

For the intra5, intra15 and post-stimulation time periods, the five ensemble models (global, hard, me, train, uni) converged to produce similar accuracies: intra5 (93.6%), intra15 (90.0%), and post-stimulation (93.8%) (Supplementary Table 1).

**Figure 2:**
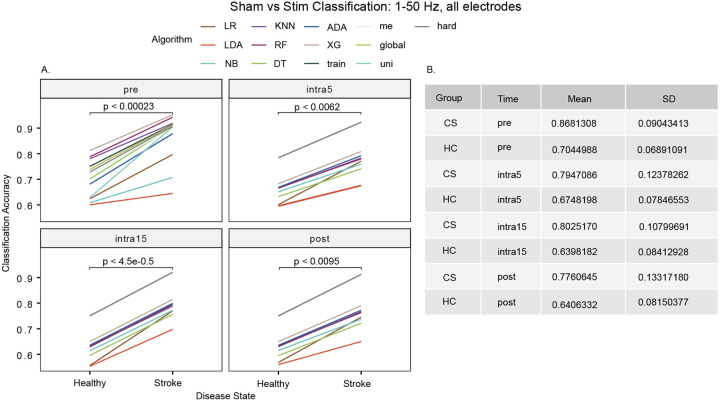
Classification of stimulation state using all frequencies 1–50 Hz and all electrodes. Classification accuracy was improved in participants with stroke across all stimulation time periods (figure 2a), in accordance with the mean accuracies shown in figure 2b. This is likely due to hemispheric asymmetry in the post-stroke brain introduced by the lesion and subsequent neuroplastic changes.

To investigate the baseline model accuracy in discriminating between sham and stimulation states, as a control we compared each algorithm using all electrodes, frequency bands, and movement states grouped together. We observed that the mean accuracy of classifying stim versus sham state was 70.4% for the HC group and 86.8% for the CS group during the pre-stimulation time period (p < 0.00023). A higher classification accuracy for the sham versus stim groups persisted throughout all time periods and was greatest at the intra15 time period (CS: 80.3 ± 10.8% versus HC: 64.0 ± 8.4%; t[_24_] = 5.4557, p < 4.5e-5, Fig. 2).

For the intra5, intra15 and post-stimulation time periods, the five ensemble models (global, hard, me, train, uni) converged to produce similar accuracies: intra5 (92.3%), intra15 (92.0%), and post-stimulation (92.0%) (Supplementary Table 2).

**Figure 3:**
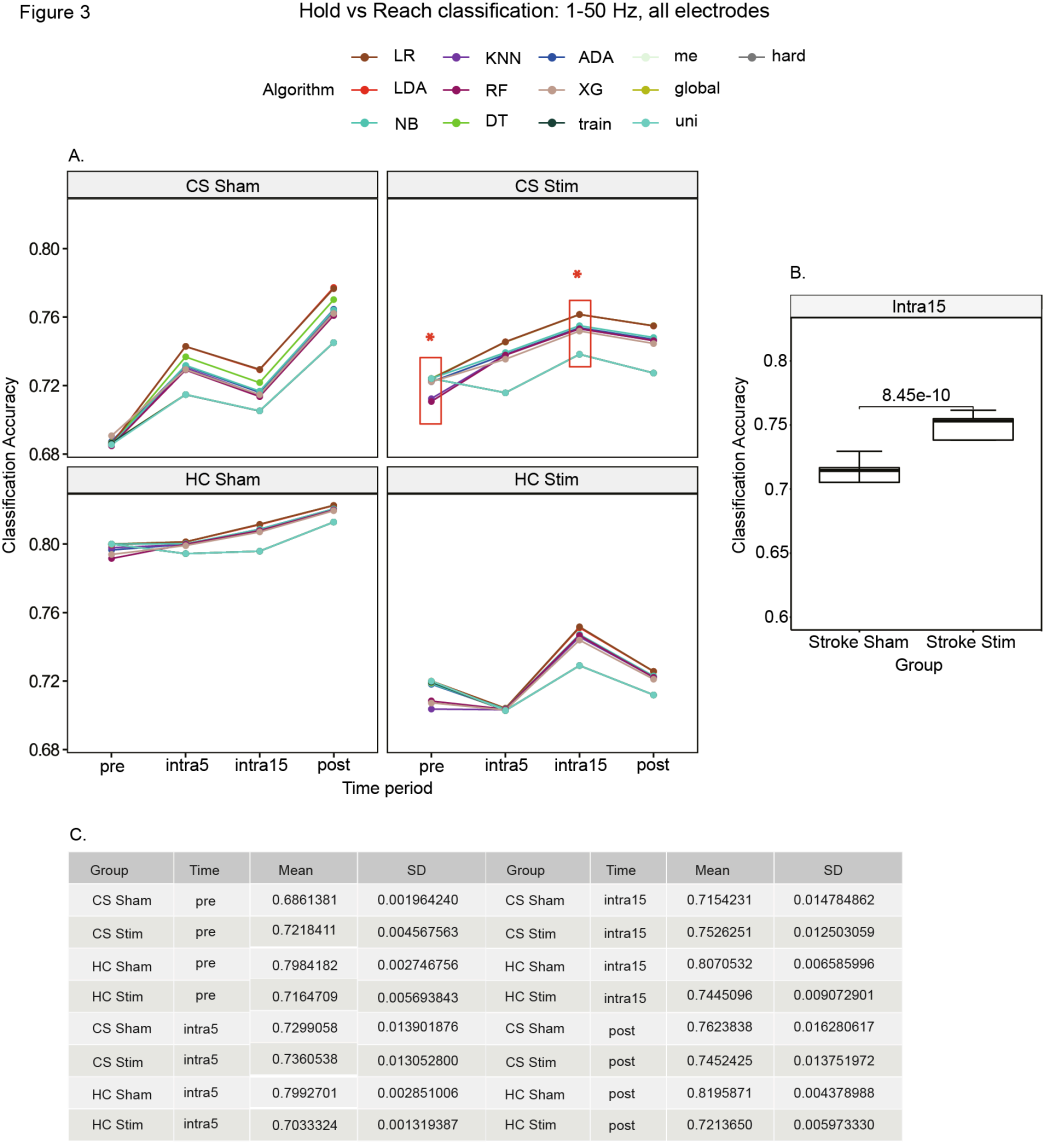
Classification of stimulation state using all frequencies 1–50 Hz and all electrodes. Classification accuracy was improved in participants with stroke across all stimulation time periods (figure 2a), in accordance with the mean accuracies shown in figure 2b. This is likely due to hemispheric asymmetry in the post-stroke brain introduced by the lesion and subsequent neuroplastic changes.

To investigate the accuracy of each algorithm in discriminating between hold and reach movement states, we created models using all electrodes and frequency bands (Fig. 3). We observed that the mean accuracy of classifying hold versus reach was 68.6 ± 0.2% for the CS sham group, 72.2 ± 0.5% for the CS stim group, 79.6 ± 0.3% for the HC sham group, and 71.6 ± 0.6% for the HC stim group at the pre-stimulation time period (Fig. 3C). A higher mean classification accuracy for sham groups persisted throughout all time periods except for the CS cohorts at the pre and intra15 time periods. At the intra15 time period, the mean accuracy for classifying hold versus reach was 75.3 ± 1.3% for the CS stim group and 71.5 ± 1.5% for the CS sham group (t[_23_] = 9.7250; p = 8.45e-10, Fig. 3, Supplemental Table 3).

In the CS stim group, LR, LDA, and DT each performed this classification with 76.1% accuracy at the shortest average training time of 0.77 sec per model (lines superimposed in Fig. 3). By comparison, XGBoost performed this classification with 75.2% accuracy at the longest average training time of 1 minute 3.8 seconds sec per model.

**Figure 4:**
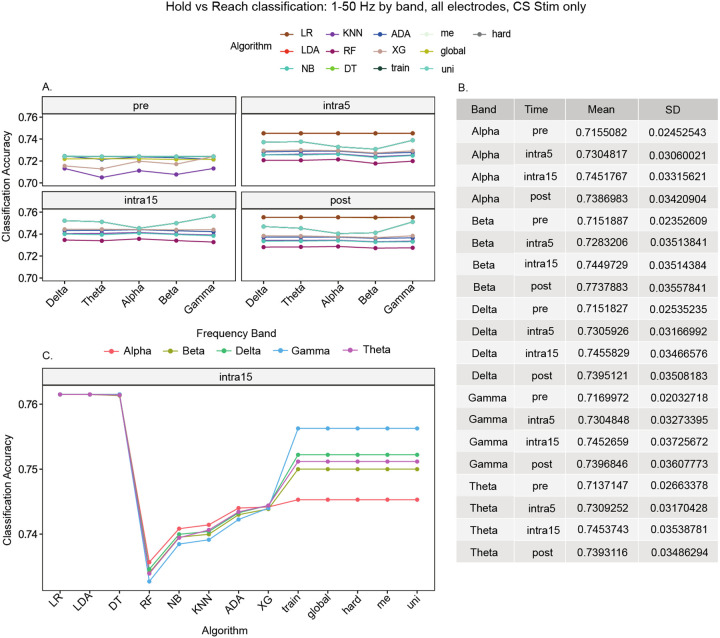
Hold versus reach movement classification using all frequencies 1–50 Hz and all electrodes. We observed the largest difference in accuracy after stimulation in participants with stroke at the intra15 time period. LR, LDA, and DT (superimposed in CS Stim subplot) produced the highest (equal) accuracies at this time.

To investigate the accuracy of each algorithm in discriminating between hold and reach movement states by frequency band, we created models using all electrodes recorded in the CS stim group. We observed that the mean accuracy of classifying hold versus reach was consistently higher in the stimulation and post-stimulation time periods (Fig. 4A, note that for the pre-stimulation period, RF classification accuracy was below 65% for all frequency bands and is not shown). Interestingly, 10 out of 13 algorithms showed no differences in accuracy between frequency bands. At the intra15 time period, LR, LDA, and DT classified hold versus reach equally at the highest accuracy (76.1%, Fig. 4B) for all frequency bands.

In contrast, the five ensemble models (global, hard, me, train, uni) showed the highest classification accuracy for the gamma frequency band at the intra15 time period in comparison to all other bands (alpha = 74.5%, beta = 75.0%, theta = 75.1%, delta = 75.2%, and gamma = 75.6%; Supplementary Table 4), although this difference was not statistically significant (p = 0.37).

**Figure 5:**
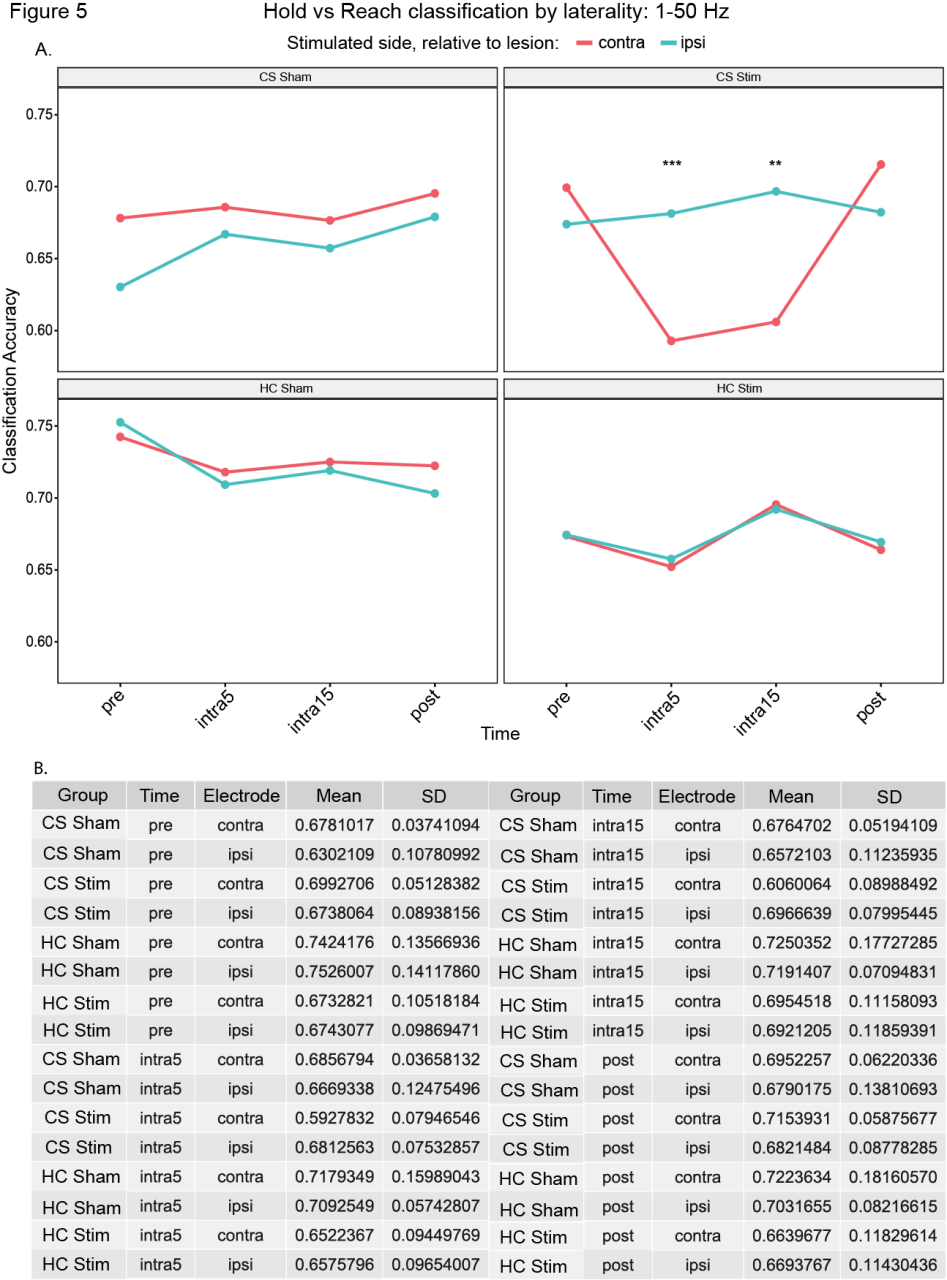
Hold versus reach movement classification using C3 or C4 and all frequencies 1–50 Hz. Here we investigated the effect of the laterality of the recording electrode relative to the lesion, where ipsi-lesional is defined as the electrode positioned on the side of the lesion. We observed that contralesional classification accuracy drops with stimulation in participants with stroke. That this pattern is not reproduced in healthy controls when stimulated suggests that this is not due to artifact, and hints at neuroplastic adaptation in the relationship of both hemispheres that occurs secondary to stroke.

To investigate the accuracy of each algorithm in discriminating between hold and reach states by electrode laterality, we created models using all frequency bands. We chose the electrode overlying primary motor cortex (C3 and C4) according to the contralateral hand used to perform the reaching task. That is, if the right hand performed the task, then the C3 electrode was labeled as the ipsi-stimulated electrode while the C4 electrode was labeled as the contra-stimulated electrode and vice versa. We observed that the mean accuracy of classifying hold versus reach was consistently higher in the contra-stimulated electrode for the sham groups (i.e., HC and CS) except for the pre-stimulation period in the HC sham cohort. In contrast, in the CS stim group, classification accuracy was highest in the ipsi-stimulated electrode during the stimulation periods only (intra 5: t_[29]_=−4.26, p = 0.0003; intra1 5: t_[23]_=^−^3.72, p = 0.0011, Fig. 5A, Supplemental Table 5).

Notably, the classification accuracy was similar in both the contra-stimulated and ipsi-stimulated electrodes in the HC stim group, suggesting that the higher accuracy in the ipsi-stimulated electrode in the CS stim group is likely not driven purely by stimulation artifact. Moreover, the classification accuracy in the ipsi-stimulated electrode in the CS stim group is constant relative to the pre-stimulation state, again suggesting a physiological response to stimulation that peaks at the intra15 time period (Fig. 5A, Supplemental Table 5).

## Discussion

As neuromodulation becomes an accepted adjunct for chronic stroke recovery, the potential use of brain stimulation to assist in identifying movement states from brain recordings has become a topic of great interest. Detection of movement intention using EEG recordings has been successfully performed in several studies using healthy and tetraplegic participants. However, real movement classification in individuals with hemiparesis is not well understood, including the dimensionality involved in modeling relevant parameters. Moreover, in implanted brain recording and stimulation systems, compute power is limited to the onboard processor, which prohibits the typical types of algorithms employed, e.g., deep learning and other neural network-based strategies. To surpass these constraints, using tuning optimized for biosignals, supervised ML approaches can be used to model high-dimensional parameter sets in order to arrive at acceptable accuracies and modeling times for control signal classification. The central problem addressed in this study is whether movement classification based on EEG recordings is possible (i.e., above chance) in chronic stroke survivors undergoing NIBS during performance of a functional task. The dataset used in this study was obtained from 10 chronic stroke survivors and 11 healthy control participants. Each group contained both active and sham stimulation cohorts. All participants followed a cued virtual reality (VR) arm reaching task, which was repeated before, during and following stimulation. EEG was recorded throughout the entire experiment. Our overall finding is that NIBS improved classification accuracy primarily in the chronic stroke group with a preference for gamma frequency band in the ensemble methods.

Comparing our results to similar studies that developed EEG classifiers for movement states, SVM performed modestly better in 46.4% of models than LDA and LR in classifying rest, simple arm movement, goal-oriented arm movement and hand clenching using motor imagery.^33^ In our study, the performance of LDA for hold versus reach (at 76.1% accuracy during the intra15 time period) was within range of the accuracies reported by Yong and Menon^33^ (75–81% accuracy) and higher than those of Rodrigo et. al.^34^ (64–68% accuracy). The voting classifiers trained in this study for hold versus reach in the chronic stroke cohort (at the intra15 time period, see Table 3) achieved an accuracy of 73.8%, which is not as strong as the voting classifier created by Khrishna et. al.^35^ (86% accuracy). Similar to the dataset used by Mebarkia and Reffad,^13^ the dataset used by Khrishna et. al.^35^ classifies motor imagery in right arm, left arm, right foot, and left foot without any consideration for hold or preparation, which may explain the stronger performance. The AdaBoost classifier trained as part of our study achieved an accuracy of 75.3% using decision trees as the classifier base, which is comparable to the AdaBoost classifiers trained by Gao et. al.^28^ who used SVM and LDA bases to achieve an accuracy of 74% and 72%, respectively.

A few studies have combined tDCS and BCI device technology with mixed results. Matsumoto et al.^36^ used a motor imagery (MI) BCI in concert with multiple 1 mA 10 min tDCS sessions in 6 healthy participants. In their investigation, mu ERD improved with anodal and attenuated with cathodal stimulation.^36^ Kasashima and colleagues^37^ repeated the paradigm in participants with stroke and hemiparesis, demonstrating similar results. In Wei et al’s^38^ study, tDCS specifically modulated upper mu (10–14 Hz) and beta (14–26 Hz) frequencies in 32 healthy controls. Hong and colleagues^39^ introduced diffusion and perfusion MRI following tDCS in combination with MI-BCI. Tractography estimates showed significant changes on the ipsilesional side for participants receiving tDCS, though no difference in motor improvement was observed between stim and sham groups.^39^ Interestingly, the authors also showed bilateral changes in parietal cerebral blood flow correlating with functional recovery and were one of the first to suggest an interhemispheric mechanism driving tDCS/MI-BCI function.^39^ Notwithstanding, tDCS did not appear to influence MI-BCI performance in a randomized, double-blinded controlled trial in 19 participants with stroke.^40^ These results did not differ from a subsequent study in which functional MRI was used to derive a low-frequency fluctuation metric.^41^ None of the studies outlined utilized real movements as a control.

Although we observed that the ensemble methods achieved the highest hold versus reach classification using gamma power as a feature, modulation of gamma in individuals with stroke is only sparsely reported. In Tecchio et al’s^42^ study, increased gamma power (33.5–44 Hz) in the affected hemisphere of chronic stroke participants was correlated with motor improvement using magnetoencephalography recordings. Moreover, Pellegrino and colleagues^43^ demonstrated that gamma reactivity to an auditory stimulus in chronic stroke participants was tightly correlated to clinical outcome as measured by Barthel Index and Functional Independence Measure. Yet in a recent systematic analysis of randomized controlled trials examining the utility of BCIs in stroke motor recovery, gamma power was absent from the frequency bands investigated as a potential biomarker.^6^

### Limitations

This study was not without limitations. First, a small sample size limits interpretability of our classification results. Second, we observed significant differences in mean age between our chronic stroke (63.3 ± 10.2 years) and healthy (46.3 ± 11.3 years) cohorts, potentially confounding our observed differences in classification accuracy which may have been affected by age-related changes in the brain. Third, to attempt to mimic the processing and bit rate constraints of a fully implantable BCI system,^44^ we avoided training more comprehensive deep learning models. Nevertheless, a robust classification pipeline using Convolutional Neural Networks was described by Lun et. al.^45^ who trained a 5-layer model on the Physionet database.^46,47^ Remarkably, using only 10 participants from that dataset, a global accuracy of 94% or above was demonstrated. Similar to our study, they limited pre-processing of the EEG signal and still achieved high classification accuracies.^45^ In our study, training was limited to 12 repetitions per task and four tasks per participant. Furthermore, results are almost certainly confounded by some proportion of learning of the task, though we attempted to limit this by randomizing several features, including time between movement states and location of reach target. The effect of learning on our results should be mitigated by the controls that were incorporated, including healthy participants and sham stimulation. We limited the parameter space explored to aspects of the task design, and we have not carried out a full examination of the preprocessing and feature extraction components of the ML pipeline. For example, Chen et al.^48^ used differential entropy to improve classification performance in EEG recordings during a cognitive task. Finally, we did not include asymmetry index measures for the chronic stroke participants as some authors have in order not to bias the comparisons to healthy participants.^15^ Given the heterogeneity of each chronic stroke participant, the extent to which each of these factors contributes to the need to personalize the training models for each individual should be explored further in future studies.

## Conclusions

Machine learning models were able to classify movement state in participants with chronic stroke using EEG even with minimal preprocessing. Classification accuracy was improved with tDCS in these participants, and the highest accuracies were obtained during stimulation rather than post-stimulation. These findings portend a brain-computer interface which can auto-detect movement state and deliver therapeutic stimulation when most beneficial for chronic stroke rehabilitation.

## Data Availability

The data, as well as code, that support the findings of this study are available on request from the lead author.

## Abbreviations

ADA: adaptive boost
CS: chronic stroke
DT: decision tree
EEG: electroencephalogram
Global: soft-vote ensemble weighted based on overall accuracy
Hard: hard vote ensemble
KNN: k-nearest neighbors
LDA: linear discriminant analysis
LR: logistic regression
Me: Soft-vote ensemble with weights chosen based on the mean of multiple base estimators
NB: naive bayes
RF: random forest
Stim: stimulation
tDCS: transcranial direct current stimulation
Train: Soft-vote ensemble weighted based on training performance
Uni: soft-vote ensemble weighted equally
VR: virtual reality
XG: XGboost
